# Risk factors for viral hepatitis C infection in Rwanda: results from a nationwide screening program

**DOI:** 10.1186/s12879-019-4322-7

**Published:** 2019-08-05

**Authors:** Jean Damascene Makuza, Carol Y. Liu, Corneille Killy Ntihabose, Donatha Dushimiyimana, Sabine Umuraza, Marie Paul Nisingizwe, Justine Umutesi, Janvier Serumondo, Soline Dusabeyesu Mugeni, Muhamed Semakula, Neil Gupta, Margaret Hellard, Sabin Nsanzimana

**Affiliations:** 1 IHDPC Department, Rwanda Biomedical Center, Po Box 7162, Kigali, Rwanda; 2 Health Department, Clinton Health Access Initiative (CHAI), Kigali, Rwanda; 3 School of Population and Public Health, University of British Columbia, Vancouver, Canada; 4 Division of Global Health Equity, Brigham & Women’s Hospital, Boston, USA; 5 Department of Infectious Disease at the Alfred Hospital and Infectious Diseases Epidemiology, Monash University in Melbourne, Melbourne, Australia

**Keywords:** Viral hepatitis C, Risk factors, Rwanda

## Abstract

**Background:**

The epidemiology and risk factors for hepatitis C virus (HCV) infection in Rwanda are not well known; however, this information is crucial to shaping the country’s public health approach to hepatitis C control.

**Methods:**

A HCV screening campaign was conducted in the general population in 24 districts previously identified to have a high HCV disease burden. At the time of sample collection, sociodemographic information and self-reported risk factors were collected. Bivariate and multivariate logistic regressions were conducted to assess risk factors independently associated with hepatitis C antibodies (HCVAb) seroprevalence.

**Results:**

Out of a total of 326,263 individuals screened for HCVAb, 22,183 (6.8%) were positive. In multivariate analysis, risk factors identified as statistically associated with HCVAb Seroprevalence include history of traditional operation or scarification (OR = 1.09, 95% CI: 1.05–1.14), presence of viral hepatitis in the family (OR = 1.27, 95% CI: 1.15–1.40), widowed or separated/divorced (OR = 1.36, 95% CI: 1.26–1.47), Southern province (OR = 1.98, 95% CI: 1.88–2.08) and aged 65 years and older (OR = 4.86, 95% CI: 4.62–5.11). Ubudehe category 3 (OR = 0.97, 95% CI: 0.93–1.01) and participants using RAMA (Health insurances for employees of public and private sectors) insurance (OR = 0.76, 95% CI: 0.70–0.85) had lower odds of HCV seroprevalence.

**Conclusions:**

Our findings provide important information for Rwanda’s strategy on prevention and case-finding. Future prevention interventions should aim to reduce transmission through targeted messaging around traditional healing practices and case-finding targeting individuals with a history of exposure or advanced age.

## Background

Globally, an estimated 71 million people are infected with chronic hepatitis C virus (HCV) infection [1]. Viral hepatitis contributed to 1.34 million deaths in 2015, a number comparable to annual deaths caused by tuberculosis and exceeding annual deaths caused by HIV. HCV accounts for around 400,000 deaths per year [2] and HCV-associated deaths in 2015 were mainly caused by chronic liver disease such as decompensated cirrhosis and liver cancer. The overall global HCV prevalence is estimated to be 2.5% and around 2.9% in Africa [3].

While HCV is increasingly highlighted as an important contributor to disease burden in high-income countries such as Europe, Canada and the United States [4], the burden in the African region is less known and thought to be highly variable across geographic area [5]. The prevalence of HCV among the general population in Sub-Saharan African (SSA) ranges from 0.1 to 17.5%, with countries such as Burundi (11.3%) and Cameroon (13.8%) among some of the countries with the highest prevalence in the world [6]. While increasing resources have been dedicated to address the burden of HCV in some high-income countries, to date, there remains a lack of strategic planning for prevention and management of HCV in SSA despite accumulating evidence of a significant disease burden [5]. The lack of a coordinated response among countries in SSA has further led to uncertainties on HCV prevalence and its variations across sociodemographic and geographic factors. Moreover, few studies in SSA have quantified the prevalence of past-exposures to known risk factors. The association between such risk factors and HCV infection and those studies were conducted only on specific groups, such as people living with HIV and MSM [7, 8] rather than the general population.

In Rwanda, the prevalence of HCV is not well known among the general population. Recent studies conducted in specific population groups have found the prevalence of anti-HCV (HCVAb), a marker for exposure to HCV, to be between 4.3–4.7% among people living with HIV (PLHIV) and 2.6% among pregnant women [9, 10]. Among these studies none have assessed risk factors for HCV in Rwanda.

In addition to uncertainties around HCVAb prevalence, risk factors for HCV infection in Rwanda have not been quantified on a national scale. Globally, older age, occupational risk of and being exposed to blood productsor individuals exposed to body piercings were shown to be risk factors for HCV [10–13]. In Africa, a systematic review yielded a wide range of high risk populations including, individuals infected with HIV, patients on hemodialysis, patients with history of blood transfusions, health care workers after needle stick injuries and sexually active adults with multiple partners [6].

Rwanda recently announced an ambitious campaign to eliminate HCV. Understanding the HCV prevalence and current drivers of transmission will be crucial to guiding more efficient screening campaigns and implementing preventative activities to reduce population exposure to major routes of transmission. This study reports on the results from an HCV screening campaign conducted by the Government of Rwanda including the risk factors most likely contributing to the HCV Ab Seroprevalence in Rwanda.

## Methods

### Study design

The study consists of a secondary analysis of cross-sectional data collected during the 2018 screening campaign for HCV. All data used for analysis were collected at the time of viral hepatitis screening through use of a standardized laboratory request form which contained sociodemographic, comorbidities and viral hepatitis risk factors characteristics.

### Study population and recruitment of participants

In response to the global and regional urgency to address HCV and improve understanding around HCV epidemiology, the Rwanda Biomedical Center (RBC) and partners prepared screening campaigns to identify infected patients to be linked to a free of charge treatment. In 2018, campaigns were specifically conducted in districts previously found to have higher prevalence of HCV among the general population and targeted individuals aged 25 years old and above from districts in Southern, Eastern and Northern Provinces previously found to have higher prevalence. The study population consists of voluntary participants of the 2018 campaigns. People who were not Rwandan by nationality or under 25 years old were excluded. Screening campaigns were conducted between March to October 2018 for 2 weeks in each district. Community awareness for viral hepatitis screening was done by radio advertisements and with the help of community health workers before and during the screening period. Individuals who belonged to targeted demographic groups and attended screening sites (health centers) were screened for HCV and included in the study population of this analysis.

### Data collection procedures

Trained nurses and laboratory technicians, using a laboratory request form to record demographic, clinical, and behavior characteristics, collected samples after verbal consent of participants. Unlike previous campaigns, the 2018 campaign asked individuals about their exposure to a list of known risk factors for HCV including history of undergoing health treatments such as blood transfusions, surgical interventions and traditional operations or scarifications and comorbidities such as TB, diabetes and cancer. Testing was performed at 13 sites across Rwanda using Murex enzyme-linked immunosorbent assays (ELISA) for HCVAb (version 4.0; DiaSorin S.p.A, Italy). A team of laboratory technicians from the National Reference Laboratory supervised all testing activities. When laboratory results were available, the results and the contents of the lab request form were entered into an encrypted database (Microsoft Excel). The database was de-identified for the present study and no persons involved in the analysis of data were able to access the linked database.

### Variables

The primary outcome of interest was HCVAb seroprevalence; it was a binary variable of either positive or negative as determined by the ELISA test. Independent variables included age. Age was considered as continuous variable during analysis then categorized into five groups: less than 35 years old, 35–44 years old, 45–54 years old, 55–64 years old and over 65 years old. Then sex, screening district, marital status and socioeconomic status (Ubudehe category). Ubudehe is a development programme whereby citizens are placed into different socioeconomic categories. The lowest socioeconomic category is 1 and the highest is 4. Socioeconomic status was defined in accordance with the updated Ubudehe category by the Ministry of Local Government of Rwanda (MINALOC) [14]. The households that consume less than Rwandan Francs (RWF) 105,064 [equivalent to USD 120] per year are classified in Ubudehe Category 1 (poorest category), which includes people without houses and those affected by food insecurity. Households that consume between RWF 105,064 and RWF 159,375 (exclusively) [equivalent to USD 180] per year are in Ubudehe Category 2, which includes households subsisting on manual labor or temporary employment and those capable of renting or owning their own houses. Categories 1 and 2 receive government assistance. Households that consume more than or equal to RWF 159,375 are classified in Ubudehe Category 3 and 4 [15]. These categories are not considered poor and do not receive government assistance. Category 3 includes wealthy farmers and investors while Category 4 includes large business owners and highly ranked government officials [16]. For health insurance, only community-based health insurance (Mutuelle), la Rwandaise Assurance Maladie (RAMA), and Medical Military Insurance (MMI) were categorized separately; all other insurances were categorized as “private insurances”. The RAMA health insurance is a privately operated health insurance available to individuals formally employed in the both the public and private sectors. Marital status was categorized into three groups of married, single and widowed, separated or divorced. HIV status as well as comorbidities and exposures to HCV risk factors were self-reported. Self-reported comorbidities assessed included high blood pressure (HBP), diabetes, chronic renal failure (CRF), cancer, tuberculosis (TB). Risk factors assessed that were associated with parenteral routes of transmission included history of health facility-based surgical operation, traditional surgical operation and transfusion. Traditional surgical operation practices are defined as scarifications, male circumcision, tattoos, traditional dental extraction or uvulectomy done by a community member or traditional practitioner. Other risk factors assessed were number of sexual partners and self-reported presence of diagnosed viral hepatitis in a family member.

### Statistical methods & data analysis

Data was de-identified and after data cleaning, data were transferred and analyzed using SPSS version 20.0. Pearson Chi-square tests were used to test for association between HCVAb seroprevalence and other categorical or binary variables. Potential determinants of HCV infection were assessed in bivariate and multivariable models using logistic regressions with HCVAb seroprevalence as the outcome variable. Multivariate analysis was used to determine socio-demographic, behavioral and clinical factors that were independently associated with HCVAb Seroprevalence.

All variables in bivariate analyses were considered for inclusion in multivariate regression model if their inclusion was conceptually logical. Variables that were not significant were eliminated using backward stepwise method, variables were removed based on *P*-value producing a final model that could determine independent association between variables and HCV infection. Interaction between age and comorbidities were checked in the final multivariate model.

### Ethics

The RBC of HIV/AIDS, STIs and Other Blood Borne Infections maintains the routinely collected program data analyzed for this study. The Medical Research Council of Rwanda governed the ethical procedures for the collection of these data and we obtained the authorization from the Ministry of Health to host the activities at different sites. The approval for utilization of data was obtained by Rwanda Biomedical Center (No 2407/RBC/2019).

## Results

### Study population

The total number of individuals screened was 327,383; 326,263 (99.7%) received their results of which 22,183 (6.8%) screened positive for HCVAb. There were 1120 (0.3%) individuals without results due to drop-out??, insufficient blood sample, or indeterminate result. Socio-demographic characteristics of the study population are shown in Table 1. The mean age of participants was 44.8 years. There were 145,537 (45.8%) individuals older than 45 years of age, 224,382 (68.8%) whom were female, and 247,437 (77.5%) whom were married. 149,092 (45.5%) were in Ubudehe category 3. The vast majority of participants 303,206 (93.6%) were using community-based health insurance (Mutuelle). The province with the highest number of individuals screened was Eastern Province where 110,337 (35.0%) of participants were screened.

**Table 1:** General characteristics of participants

Characteristics	Number of participants(%)	HCV Ab positive by Characteristics(%)	HCV Ab negative by Characteristics(%)
Gender(*N* = 326,175)			
Female	224,382 (68.8)	15,431 (6.9)	208,951 (93.1)
Male	101,793 (31.2)	6651 (6.6)	95,142 (93.4)
Age group(*N* = 317,431)			
< 35 years old	92,243 (29.1)	3396 (3.7)	88,847 (96.3)
35–44 years old	79,651 (25.1)	3427 (4.3)	76,224 (95.7)
45–54 years old	61,905 (19.5)	3516 (5.7)	58,389 (94.3)
55–64 years old	50,556 (15.9)	4674 (9.3)	45,882 (90.7)
65 years old and above	33,076 (10.4)	6458 (19.6)	26,618 (80.4)
Marital status(*N* = 319,302)			
Married	247,437 (77.5)	1030 (3.7)	23,713 (96.3)
Single	28,188 (8.8)	15,266 (6.2)	12,922 (93.8)
Widow, Divorced and Separated	43,677 (13.7)	4866 (11.2)	38,811 (88.8)
Ubudehe category(*N* = 318,855)			
Category 1	49,886 (15.6)	4462 (9.0)	45,424 (91.0)
Category 2	119,643 (36.5)	8034 (6.7)	111,609 (93.3)
Category 3	149,092 (45.5)	9097 (6.1)	139,995 (93.9)
Category 4	234 (0.1)	17 (7.3)	217 (92.7)
Health Insurance(*N* = 323,953)			
Mutuelle	303,206 (93.6)	21,095 (7.0)	282,111 (93.0)
RAMA	14,708 (4.5)	515 (3.5)	14,193 (96.5)
MMI	2574 (0.8)	78 (3.0)	2496 (97.0)
Other private insurances	3465 (1.1)	218 (6.3)	3247 (93.7)
Province of screening(*N* = 315,040)			
East	110,337 (35.0)	3313 (5.0)	107,024 (95.0)
North	65,824 (20.9)	5277 (5.9)	60,547 (94.1)
West	89,885 (28.5)	5880 (12.0)	84,005 (88.0)
South	48,994 (15.6)	7264 (6.6)	41,730 (93.4)

Table 2 shows self-reported clinical characteristics and historical exposures of participants. Among comorbidities, 1867 (0.6%) reported diabetes, 9510 (2.9%) reported high blood pressure (HBP), 578 (0.2%) reported chronic renal failure (CRF), 293 (0.2%) reported cancer, 6597 (2%) reported HIV-positive status, and 338 (0.1%) reported a history of tuberculosis. Among self-reported historical exposures to risk factors, 17,876 (5.6%) were surgically operated upon at least once, 5350 (1.6%) received a blood transfusion at least once, 54,097 (16.6%) had history of a traditional surgical practice, 8755 (2.7%) had multiple sex partners (either current or historical), and 6190 (1.9%) had a family member diagnosed with viral hepatitis. Among all variables, no variables had > 5% missing values.

**Table 2:** Distribution of potential self-reported risk factors for hepatitis C that were assessed in the analysis

Characteristics	Number of participants(%)	HCV Ab positive by Characteristics(%)	HCV Ab negative by Characteristics(%)
Suffering from Diabetes(*N* = 326,926)			
No	325,059 (99.4)	21,961 (6.8)	303,098 (93.2)
Yes	1867 (0.6)	182 (9.8)	1685 (91.2)
Suffering from HBP (*N* = 326,931)			
No	317,421 (97.1)	21,054 (6.7)	296,367 (93.3)
Yes	9510 (2.9)	1090 (11.5)	8420 (88.5)
Suffering from CRF(*N* = 326,930)			
No	326,323 (99.8)	21,692 (6.7)	10,940 (93.3)
Yes	578 (0.2)	452 (10.9)	126 (89.1)
Suffering from Cancer (*N* = 326,901)			
No	32,016 (99.8)	22,079 (6.8)	9937 (93.2)
Yes	293 (0.2)	63 (10.9)	230 (89.1)
HIV status (*N* = 326,913)			
Negative	320,316 (98.0)	21,673 (6.8)	298,643 (93.2)
Yes	6597 (2.0)	470 (7.1)	6127 (92.9)
Ever had TB(*N* = 326,919)			
No	326,581 (99.9)	22,108 (6.8)	304,473 (93.2)
Yes	338 (0.1)	36 (10.7)	302 (89.3)
Ever been operated(*N* = 326,913)			
No	309,037 (94.4)	20,916 (6.8)	288,121 (93.2)
Yes	17,876 (5.6)	1225 (6.9)	16,651 (93.1)
Ever been transfused(*N* = 326,922)			
No	321,572	21,691 (6.8)	299,881 (93.2)
Yes	5350 (1.6)	452 (8.5)	4898 (91.5)
Traditional operation and scarification(*N* = 326,652)			
No	272,555 (83.4)	18,047 (6.6)	254,508 (93.4)
Yes	54,097 (16.6)	4089 (7.6)	50,008 (92.4)
Having more than 1 sexual partner(*N* = 326,785)			
No	318,030 (97.3)	21,601 (6.8)	296,429 (93.2)
Yes	8755 (2.7)	538 (6.2)	8217 (93.8)
Viral Hepatitis in the family(*N* = 326,882)			
No	320,692 (98.1)	21,609 (6.8)	299,083 (93.2)
Yes	6190 (1.9)	531 (8.6)	5659 (91.4)

In the multivariate analysis as shown in Table 3, being a male was statistically associated with HCVAb Seroprevalence [adjusted-OR (aOR) = 1.06, 95%CI:(1.03–1.10)]. Age greater than 65 years old [aOR = 4.87, 95%CI:(4.62–5.11)], and widowed/ separated and divorced marital status [aOR = 1.36, 95%CI:(1.26–1.47)] were both associated with HCV Ab Seroprevalence. Having RAMA as health insurance had lower odds of HCVAb seroprevalence compared to those on Community based health insurance [aOR = 0.78, 95%CI:(0.70–0.85)] and being in Ubudehe Category 3 and 4 had lower odds of HCVAB seroprevalence than being in Ubudehe category 1 [aOR = 0.92 95%CI:(0.88–0.96)]. Relative to the Northern Province, all other provinces had a significantly higher seroprevalence in the Western Province, Southern Province and Eastern Province [aOR = 1.37, 95%CI:(1.31–1.43)]. Self-reported co-morbidities found to be associated with HCVAb prevalence included high blood pressure (aOR = 1. 95%CI:(1.13–1.31)] and CRF (aOR = 1.29, 95%CI:(1.16–1.44)]. Self-reported risk factors associated with HCVAb prevalence were history of traditional surgical practices (aOR = 1.09, 95%CI:(1.05–1.14)] and history of a family member diagnosed with viral hepatitis (aOR = 1.27, 95%CI: (1.15–1.40)].

**Table 3:** Prevalence of HCV according to different characteristics among participants

Characteristics	Number of participants	HCV positive(%)	Bivariate analysis			Multi-variable analysis		
			OR	(95%CI)	*P*-value	OR	(95%CI)	*P*-value
Gender(*N* = 325,078)								
Female	223,627	15,431 (6.9)	1			1		
Male	101,451	6651 (6.6)	0.95	(0.92–0.98)	< 0.001	1.06	(1.03–1.10)	< 0.001
Age group(*N* = 316,336)								
< 35 years old	91,922	3396 (3.7)	1			1		
35–44 years old	79,337	3427 (4.3)	1.18	(1.12–1.24)	< 0.001	1.11	(1.05–1.17)	< 0.001
45–54 years old	61,733	3516 (5.7)	1.57	(1.50–1.65)	< 0.001	1.30	(1.23–1.37)	< 0.001
55–64 years old	50,379	4674 (9.3)	2.67	(2.55–2.79)	< 0.001	2.11	(2.00–2.22)	< 0.001
65 years old and above	32,965	6458 (19.6)	6.35	(6.08–6.64)	< 0.001	4.86	(4.62–5.11)	< 0.001
Marital status (*N* = 318,211)								
Single	28,105	1030 (3.7)	1			1		
Married	246,593	15,266 (6.2)	1.74	(1.63–1.85)	< 0.001	1.152	(1.07–1.24)	< 0.001
Widow, separated and divorced	43,513	4866 (11.2)	3.31	(3.09–3.55)	< 0.001	1.36	(1.26–1.47)	< 0.001
Ubudehe category(*N* = 318,784)								
Category 1	49,767	4462 (9.0)	1			1		
Category 2	119,347	8034 (6.7)	0.73	(0.71–0.76)	< 0.001	0.97	(0.93–1.01)	0.112
Category 3 and 4	148,686	9114 (6.1)	0.66	(0.64–0.69)	< 0.001	0.92	(0.88–0.96)	< 0.001
Health Insurance(*N* = 322,856)								
Mutuelle	302,152	21,095 (7.0)	1			1		
RAMA	14,673	515 (3.5)	0.49	(0.44–0.53)	< 0.001	0.775	(0.70–0.85)	< 0.001
MMI	2570	78 (3.0)	0.42	(0.33–0.52)	< 0.001	0.804	(0.63–1.02)	0.08
Other private insurances	3436	218 (6.3)	0.90	(0.78–1.03)	0.12	1.06	(0.91–1.23)	0.47
Province of screening(*N* = 313,945)								
North	65,804	3313 (5.0)	1			1		
West	89,767	5277 (5.9)	1.18	(1.13–1.23)	< 0.001	1.19	(1.14–1.25)	< 0.001
South	48,976	5880 (12.0)	2.57	(2.46–2.69)	< 0.001	1.98	(1.88–2.08)	< 0.001
East	109,398	7264 (6.6)	1.34	(1.40–1.50)	< 0.001	1.37	(1.31–1.43)	< 0.001
Suffering from Diabetes(*N* = 325,829)								
No	323,971	21,961 (6.8)	1.00					
Yes	1858	182 (9.8)	1.49	(1.28–1.74)	< 0.001			
Suffering from HBP (*N* = 325,834)								
No	316,372	21,054 (6.7)	1.00			1.00		
Yes	9462	1090 (11.5)	1.83	(1.71–1.95)	< 0.001	1.22	(1.13–1.31)	< 0.001
Suffering from CRF(*N* = 325,833)								
No	321,698	21,692 (6.7)	1.00			1.00		
Yes	4135	452 (10.9)	1.68	(1.54–1.87)	< 0.001	1.29	(1.16–1.44)	< 0.001
Suffering from Cancer (*N* = 325,804)								
No	325,228	22,079 (6.8)	1.00					
Yes	513	63 (10.9)	1.69	(1.30–2.19)	< 0.001			
HIV status (*N* = 325,816)								
Negative	319,234	21,673 (6.8)	1.00			1		
Yes	6582	470 (7.1)	1.06	(0.96–1.16)	0.26	1.08	(1.05–1.32)	0.001
Ever had TBC(*N* = 325,822)								
No	325,484	22,108 (6.8)	1.00					
Yes	338	36 (10.7)	1.636	(1.16–2.31)	0.005			
Ever been operated(*N* = 325,816)								
No	307,980	20,916 (6.8)	1.00					
Yes	17,836	1225 (6.9)	1.01	(0.95–1.07)	0.69			
Ever been transfused(*N* = 325,825)								
No	320,488	21,691 (6.8)	1.00					
Yes	5337	452 (8.5)	1.28	(1.16–1.41)	< 0.001			
Traditional operation and scarifications (*N* = 325,555)								
No	271,590	18,047 (6.6)	1.00			1.00		
Yes	53,965	4089 (7.6)	1.15	(1.11–1.19)	< 0.001	1.09	(1.05–1.14)	< 0.001
Viral Hepatitis in the family(*N* = 325,785)								
No	319,616	21,609 (6.8)	1.00			1.00		
Yes	6169	531 (8.6)	1.30	(1.19–1.42)	< 0.001	1.27	(1.15–1.40)	< 0.001
Interaction Age_HBP(*N* = 326,263)								
< 35 years old	317,891	21,669 (6.4)	1			1		
35–44 years old	1418	80 (5.6)	0.84	(0.67–1.05)	0.12	1.14	(0.80–1.61)	0.46
45–54 years old	1880	111 (5.9)	0.88	(0.73–1.07)	0.19	1.27	(1.01–1.61)	0.05
55–64 years old	2606	276 (10.6)	1.66	(1.46–1.83)	< 0.001	1.04	(0.85–1.27)	0.71
65 years old and above	2468	574 (22.2)	3.99	(3.62–4.39)	< 0.001	1.18	(1.06–1.30)	0.002
Interaction Age _CRF(*N* = 326,263)								
< 35 years old	322,909	21,788 (3.7)	1			1		
35–44 years old	767	48 (6.3)	0.13	(0.09–0.20)	< 0.001	1.32	(0.97–1.81)	0.08
45–54 years old	765	56 (7.3)	0.21	(0.15–0.29)	< 0.001	1.38	(1.01–1.61)	0.03
55–64 years old	1069	129 (12.1)	0.25	(0.18–0.34)	< 0.001	1.26	(1.04–1.54)	0.02
65 years old and above	753	183 (24.3)	0.43	(0.33–0.55)	< 0.001	1.24	(1.03–1.50)	0.02
Interaction Age _HIV(*N* = 326,263)								
< 35 years old	321,031	21,801 (6.8)	1			1		
35–44 years old	1940	119 (6.1)	1.00	(0.80–1.26)	0.97	1.36	(1.12–1.65)	0.00
45–54 years old	1920	101 (5.3)	0.76	(0.62–0.93)	0.01	0.89	(0.73–1.10)	0.29
55–64 years old	1023	92 (9.0)	1.36	(1.09–1.68)	0.01	0.93	(0.75–1.16)	0.53
65 years old and above	349	70 (20.1)	3.44	(2.65–4.48)	< 0.001	1.01	(0.77–1.32)	0.94

## Discussion

This study is the first national-level study in Rwanda to assess risk factors for HCV using the country’s seroprevalence survey for HCV exposure (HCVAb). The national-level coverage and large sample size contribute to the strength of our findings. To our knowledge this is the first study to assess risk factors associated with HCVAb prevalence in a nationally-representative screening program for members of the general population in SSA.

Compared to previous studies conducted in Rwanda among PLHIV and pregnant women that produced prevalence estimates of 4.6 and 2.6% respectively [9, 10], the HCVAb seroprevalence estimated by this study is 6.8%. The likely explanation for this higher prevalence is the strategic decision by the campaign to target older individuals and the self-selected nature of voluntary participants where individuals who had reason to suspect that they had viral hepatitis may have come forward for testing. Factors associated with being HCVAb positive following adjusted analysis included older age with 0.1% of risk of developing HCV every year for each participant, lower socioeconomic status, geographic variation, family history of HCV and exposures to traditional surgical operations. Associations between HCV infection and both family history of HCV and exposures to traditional surgical operations remained significant after adjusting for age.

Traditional scarifications and operations, though heavily discouraged, are still widely prevalent in informal healthcare practices in Rwanda. Although data on frequency of traditional surgical practices in Rwanda is limited, the Rwanda Demographic Health Survey 2015 reported that 8.5% of circumcisions for people between 15 and 59 years old were performed by traditional practitioners or a family friend [17]. Collectively, these findings suggest a need for interventions targeted to traditional healers such as increasing population awareness on the risks of traditional cuttings for infectious disease transmission. The Ministry of Health and RBC strongly advise individuals in Rwanda to seek health advice from official health facilities rather than traditional healers. Our findings add further evidence to the potential harm of unregulated traditional practices.

Although the most frequently discussed transmission routes for HCV are parenteral, there has also been much debate on the impact of household exposure on HCV transmission [18]. Our study found an association between familial history of viral hepatitis and HCVAb prevalence. Previous observational studies have reported clustering of cases within households and documented evidence of higher disease prevalence among individuals with an infected family member compared to the general population [19–22]. Moreover, a recent cross-sectional survey of HCV patients conducted in China showed that long term exposure to an infected family member was associated with infection [23], an indication that a constant exposure to low-risk transmission routes such as razors, tooth brushes and nail clippers could still contribute to infection. However, another plausible explanation for our findings is that members of the same household could be exposed to the same external risk factors. For example, family members visiting the same traditional medicine practitioner with unsafe needle practices may have an elevated risk for infection. Given low awareness among the population on potential risk of transmission of HCV within households, patients with HCV should be counselled on prevention of disease transmission to their cohabitants. Cohabitants should be offered the option of receiving HCV counseling and undergoing HCV testing.

**Fig. 1:**
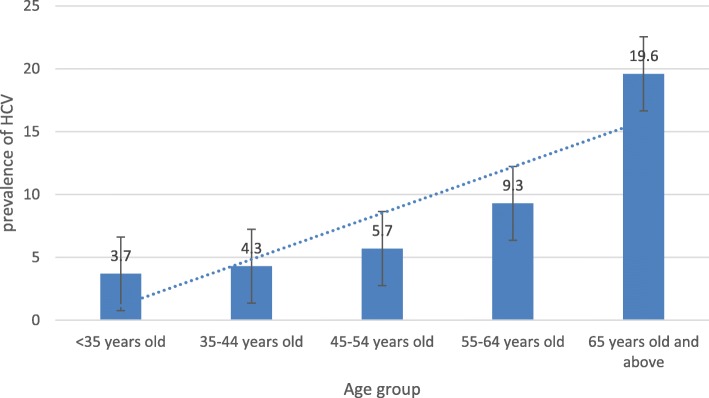
Prevalence of HCVAb by age category of participants

Similar to other studies conducted in Rwanda on people living with HIV and on pregnant women [9, 10], older age showed strong associations with HCVAb with a trend of higher odds of infection with increasing age group (Fig. 1). It is likely that older individuals are more likely to have historical exposure to risk factors such as unhygienic medical procedures and scarifications, either within a health facility or with traditional practitioners, prior to implementation of current infection control policies.

Co-morbidities that showed associations with HCVAb prevalence were HBP and CRF. According a large household survey in Rwanda, 33.2% of people aged 55–64 years old had high blood pressure while 6.7% of people between 25 and 34 years old had high blood pressure [24]. There might be interaction between co-morbidities and age (see Table 3). CRF could be a complication of chronic HCV and HCV can be acquired during different procedures performed by health care providers during management of CRF, such as dialysis [25]. Self-reported HIV infection was associated with HCVAb prevalence and can be explained by potential shared modes of transmission between HIV and HCV.

Individuals in socioeconomic category of Ubudehe category 3 have lower odds of HCVAb compared to individuals in the lowest socioeconomic category of Ubuduhe category 1. Individuals using RAMA as health insurance compared to those using Mutuelle also have lower odds of infection. Individuals from lower socioeconomic categories may be at a higher risk for HCV due to more exposures to unhygienic practices in informal health settings or sharing of sharps such as razors.

This study also found that people from the Northern province have lower Seroprevalence for HCV compared to other provinces. This may be due to the fact that other provinces have higher migration across borders and a greater refugee population. Apart from socio-economic status, other unmeasured cultural practices or risk factors could have contributed to these geographical differences [10, 11, 13, 23, 26].

Several limitations were identified. The demographic profile of the sample population of voluntary participants differed from the Rwandan population at large, with a substantially greater proportion of females (69%) and a higher median age (43.0) than the general population. Thus, the prevalence estimate and risk factors found to be associated with HCVAb may not be generalizable to the entire population. Since children are less likely to be infected with HCV, the prevalence of HCV in Rwanda is likely lower than reported in this study. As this study utilized presence of anti-HCV antibody as the primary marker of HCV infection, the risk factors identified are relevant for present or previous HCV infection and may not be associated with chronic viremic state. Other unmeasured risk factors such as exposure to mass casualties through war and conflict, sexual violence, refugee status, occupational risk like health care providers or community based traditional practices may have been more prevalent historically. Key populations, such as injection drug users and men who have sex with men, were not specifically identified or characterized in this study, though the proportion of individuals with these risk factors have been previously reported to be low in the Rwandan general population [27]. Lastly, this study relied on routinely collected data and self-report to assess clinical variables (e.g. HIV status) or historical exposure which may have led to misclassification. Participants are likely to have underreported at random with respect to the outcome due to poor recall of historical events. If a true association exists between HCV infection and variables identified in this study, then random misclassification of exposures would have led odds ratio towards the null. If exposures were recorded accurately, we would expect to obtain the true effect of the known risk factors on HCVAb. Also plausible is that individuals with lower health literacy had lower awareness of risk factors. These individuals could have had either higher risk for HCV infection due to more exposures to less hygienic health practices or lower risk for HCV due to less healthcare-seeking overall.

## Conclusion

HCV is a worldwide epidemic that can cause death and severe liver disease complications especially in low- and middle-income countries and has been identified as a public health problem in Sub-Saharan Africa. The high HCVAb seroprevalence found in this study reiterates the importance of addressing the HCV burden in both Rwanda and in sub-Saharan Africa. Screening should be universal and priority for testing and screening should be given to people who are at high risk such as older age, lower socioeconomic status, geographic variation, history of traditional surgical practices, and family exposures for both prevention and screening as Rwanda commits to achieving WHO targets of eliminating HCV by 2030.

## Data Availability

The datasets generated during the current study are not publicly available but are available from the corresponding author on reasonable request.

## Abbreviations

CI: Confidence Interval
CRF: Chronic Renal Failure
ELISA: Enzyme-Linked Immunosorbent Assays
HBV: hepatitis B virus
HCV Ab: Hepatitis C Virus Antibody
HCV: Hepatitis C Virus
HIV/AIDS: Human Immunodeficiency virus/ Acquired Immunodeficiency Diseases Syndrome
MEDIPLAN: Private insurance initiated by Insurance Company called SORAS
MINALOC: Ministry of Local Government
MMI: Military Medical Insurance
OR: Odd Ratio
PLHIV: People Living with HIV
RAMA: La Rwandaise Assurance Maladie: Health insurances for employees of public and private sectors
RBC: Rwanda Biomedical Center
SPSS: Statistical Package for the Social Sciences
TB: Tuberculosis
WHO: World Health Organization
